# Analysis of speech and swallowing and quality of life in patients undergoing subtotal glossectomy with free flap reconstruction followed by radiotherapy

**DOI:** 10.1080/23320885.2021.1884559

**Published:** 2021-02-22

**Authors:** Angelos Mantelakis, Konstantinos Vachtsevanos, Harry V. M. Spiers, Christina Gavriilidou, Stamatis Sapountzis

**Affiliations:** a Lewisham and Greenwich NHS Foundation Trust, London, United Kingdom of Great Britain and Northern Ireland; b Department of Oral and Maxillofacial Surgery, G Papanikolaou General Hospital, Aristotle University of Thessaloniki, Thessaloniki, Greece; c Manchester Royal Infirmary Oxford Road, Manchester, United Kingdom of Great Britain and Northern Ireland; d Efolikon Speech and Languange Therapy, Thessaloniki, Greece; e St Luke’s Hospital, Panorama, Greece

**Keywords:** Tongue cancer, speech, swallow, quality of life

## Abstract

Six patients (4 with post-operative radiotherapy, 2 without) were formally assessed by a speech and language therapist 12 months post-operatively. Patient-reported quality of life (QOL) was simultaneously measured. Patients treated with post-operative radiotherapy had lower overall speech comprehensibility scores, poorer swallowing function in puree and solid foods and lower overall QOL.

## Introduction

Surgical resection followed by free flap tongue reconstruction for tongue cancer carries significant morbidity, furthered by adjuvant radiotherapy (RT) [1–3]. While traditionally the emphasis is on surgical outcomes and mortality following cancer resection, quality of life (QOL) is fast becoming an equally important outcome of treatment for both patients and the healthcare team alike [4].

### Case series

**Figure 1.**
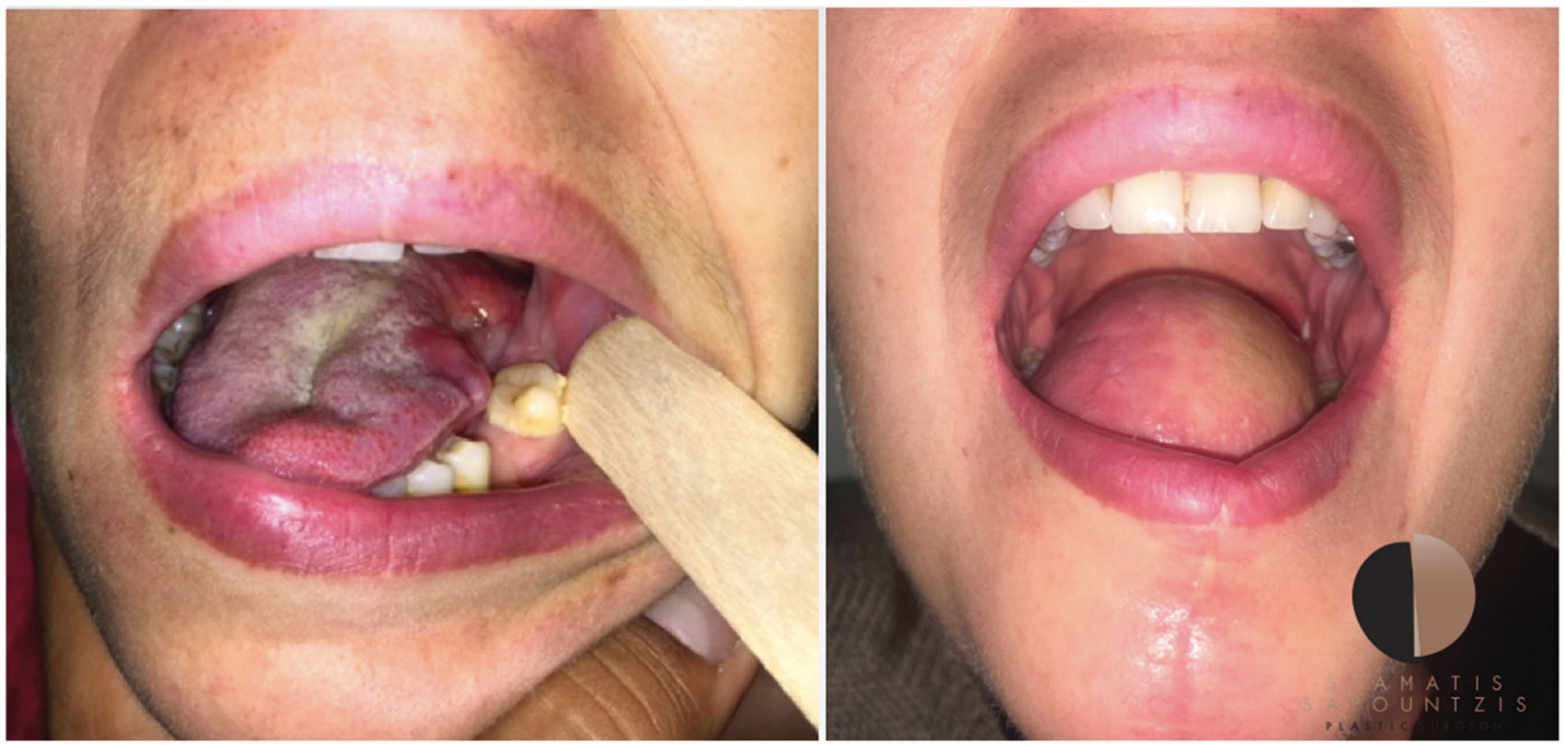
Intra-operative free-flap tongue reconstruction (a) preparation of reconstruction following hemi-glossectomy (b) the radial forearm free flap before implantation 3) pre-operative free flap design.

**Figure 2.**
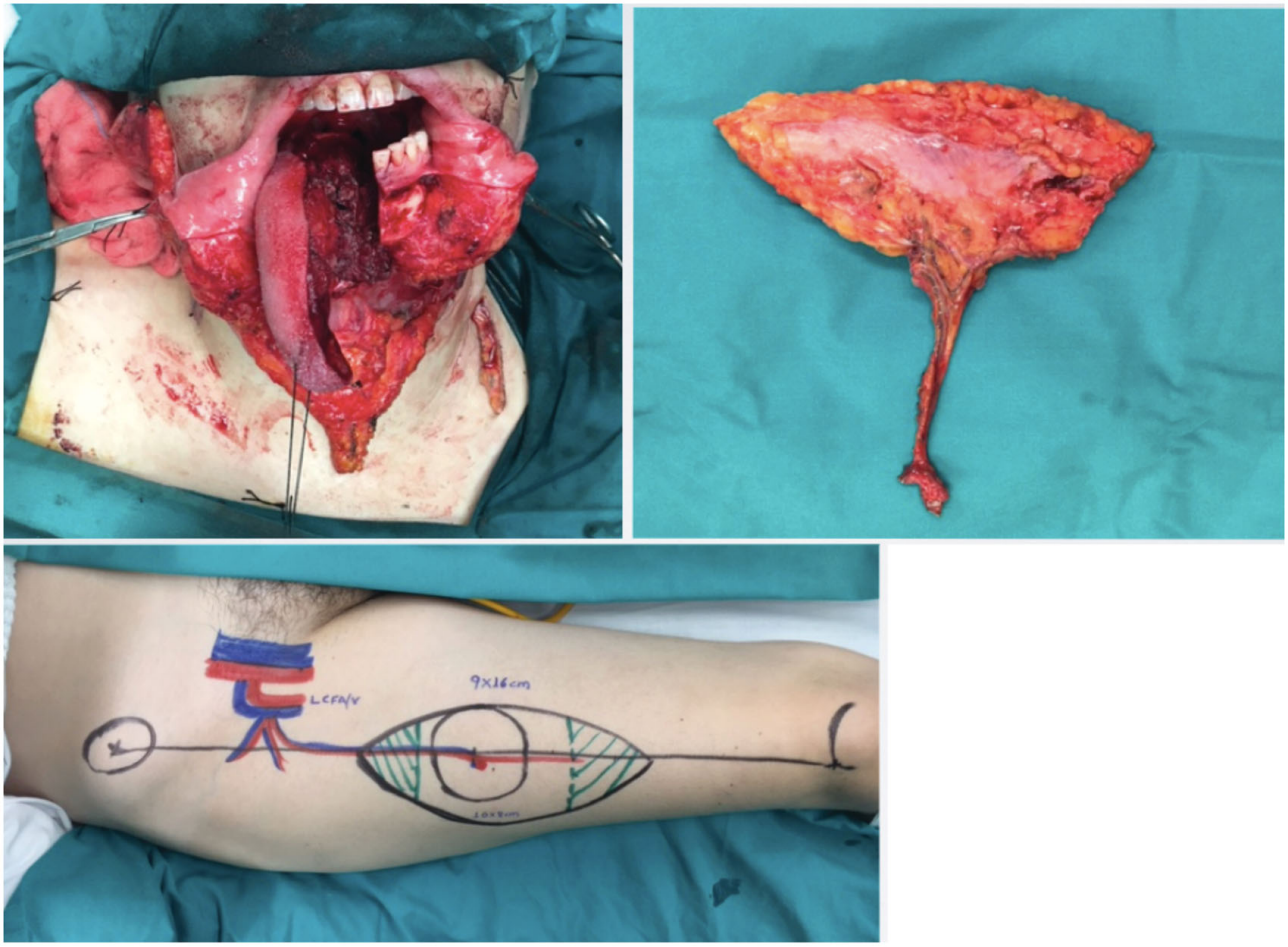
Pre-operative and post-operative outcome of tongue reconstruction utilising the radial forearm free flap.

Six patients that undertook resection with free flap reconstruction for tongue cancer, with or without radiotherapy, were recruited at 12 months post-treatment (Table 1, Figures 1 and 2). Exclusion criteria included base of the tongue resection, prior compromised speech and swallowing function, prior oral surgery, prior head and neck radiotherapy.

**Table 1.** Speech and language assessment in patients that did not undergo post-operative radiotherapy.

Patient Demographics	Radiotherapy	Speech phonetics	Speech Intelligibility	Tongue Movements	Swallowing
Patient 1 Male, 58	No	Excellent articulation, some distortion of ‘s’ vowel	90%	Present in all directions (up, down left, right), no circular movements on the right side	Excellent in liquids Some residue in puree and solids sensation intact
Patient 2 Male, 47	No	Excellent articulation	100%	Present in all directions (up, down left, right), no circular movements	Excellent in all liquids, puree and solids sensation intact
Patient 3 Female, 42	Yes	no ‘*r*’ vowel articulation, distortion of ‘*s, l, th, d’*	80%	No movement (up, down left, right), No circular movements on the right side	Excellent in liquids Some residue in pureed food without sensation Incomplete clearing of solid food with sensation
Patient 4 Female, 27	Yes	Distortion of *‘s’* only	100%	Present in all directions (up, down left, right), Some inability to elevate tongue in the upwards direction	Excellent in liquids Some incomplete clearing food during puree without sensation Incomplete clearing of solid food with sensation
Patient 5 Female, 61	Yes	absent articulation of ‘*r,l,s,z;,* distortion of ‘*d, th’*	90%	Absent in all directions (up, down left, right), No circular movements Could not protrude tongue	Excellent in liquids Some incomplete clearing food during puree without sensation Incomplete clearing of solid food with sensation
Patient 6 Female, 53	Yes	Multiple absent vowels, intelligibility severely affected	80%	Absent in all directions (up, down left, right), No circular movements Could not protrude tongue	Excellent in liquids Residue in both pure and solids, patient would require sips of water to swallow No sensation

#### Speech, language and swallowing assessment

All patients undertook a one-hour one-to-one assessment with a qualified speech and language therapist. Speech function was assessed by asking the patient to read out words and sentences from a template that use all of the vowels, diphthong tokens and alveolar sibilants. Any inability to pronounce these was recorded, and an overall speech intelligibility score was allocated (from 0% to a perfect intelligibility score of 100%)

The mobility of the tongue was examined, and swallowing function was assessed *via* the ability to swallow a food bolus completely in all three formations (liquid, pureed and solids) in the absence of residue, coughing or choking, or tearing.

In the two non-irradiated patients, an excellent or near-excellent articulation of phonetic sibilants and speech intelligibility was observed (Table 2). Tongue movements were near-normal, with the absence of the ability to perform circular movements only. Swallowing liquids was excellent in both patients, with some minor residue during pureed and solids noticed in only one of the patients.

**Table 2.** Background Information.

Patient Number, gender and age (yrs)	Histological Diagnosis	Staging (TNM classification)	Operation	Tongue defect size (% of total surface area)	Free flap reconstruction	Post-operative Radiotherapy
Patient 1 Male, 58	Squamous cell carcinoma	T3N1M0	Lt partial glossectomy and right sided lymph node clearance	40%	Radial Forearm flap	No
Patient 2 Male, 47	Squamous cell carcinoma	T2N0M0	Rt partial glossectomy and right sided lymph node clearance	40%	Radial Forearm flap	No
Patient 3 Female, 42	Squamous cell carcinoma	T3N1M0	Rt hemiglossectomy and right sided lymph node clearance and selective left sided lymph node clearance	50%	Radial Forearm flap	Yes
Patient 4 Female, 27	Squamous cell carcinoma	T4N1M0	Left partial glossectomy with right sided lymph node clearance and selective left sided lymph node clearance	40%	Radial Forearm flap	Yes
Patient 5 Female, 61	Squamous cell carcinoma	T4N0M0	Right partial glossectomy with right sided lymph node clearance	40%	Anterolateral thigh flap	Yes
Patient 6 Female, 53	Squamous cell carcinoma	T3N1M0	Left partial glossectomy with left sided lymph node clearance and selective left sided lymph node clearance	40%	Anterolateral thigh flap	Yes

**Table 3.** Patient responses to EORTC-C30 and EORTC-H&N35 questionnaires.

	Patient 1 (%)	Patient 2 (%)	Patient 3 (%)	Patient 4 (%)	Patient 5 (%)	Patient 6 (%)
Global Health Status						
Global Health Status/QoL	83.33	100.00	0.00	83.33	66.67	66.67
Functional Scales						
Physical Functioning	100.00	100.00	100.00	93.33	80.00	93.33
Role Functioning	100.00	100.00	100.00	66.67	33.33	66.67
Emotional functioning	100.00	100.00	100.00	83.33	83.33	83.33
Cognitive functioning	100.00	100.00	100.00	100.00	83.33	100.00
Social functioning	100.00	100.00	100.00	83.33	33.33	50.00
Symptom scales/items						
Fatigue	0.00	0.00	0.00	33.33	11.11	33.33
N&V	0.00	0.00	0.00	16.67	0.00	16.67
Pain	0.00	0.00	16.67	0.00	33.33	66.67
Dyspnoea	0.00	0.00	0.00	66.67	0.00	0.00
Insomnia	0.00	0.00	0.00	0.00	0.00	33.33
Appetite Loss	0.00	0.00	0.00	0.00	33.33	66.67
Constipation	0.00	0.00	0.00	100.00	66.67	0.00
Diarrhoea	0.00	0.00	0.00	0.00	0.00	0.00
Financial Difficulties	0.00	0.00	0.00	0.00	66.67	0.00
H&N35						
Pain	0.00	8.33	8.33	16.67	33.33	25.00
Swallowing	0.00	0.00	8.33	8.33	16.67	8.33
Senses problems	0.00	0.00	16.67	33.33	33.33	16.67
Speech problems	0.00	0.00	0.00	0.00	44.44	0.00
Trouble with social eating	8.33	0.00	0.00	16.67	33.33	25.00
Trouble with social contact	0.00	0.00	0.00	0.00	33.33	26.67
Less sexuality	0.00	0.00	33.33	33.33	66.67	83.33
Teeth	0.00	0.00	0.00	0.00	33.33	33.33
Opening of mouth	0.00	0.00	0.00	0.00	33.33	33.33
Dry mouth	0.00	0.00	66.67	66.67	66.67	0.00
Sticky Saliva	0.00	0.00	0.00	0.00	66.67	0.00
Coughing	0.00	0.00	0.00	0.00	0.00	0.00
Felt ill	0.00	0.00	0.00	0.00	0.00	0.00
Pain Killers	0.00	0.00	0.00	0.00	0.00	100.00
Nutritional Supplements	0.00	0.00	0.00	0.00	0.00	100.00
Feeding tube	0.00	0.00	0.00	0.00	0.00	100.00
Weight Loss	0.00	0.00	0.00	100.00	0.00	0.00
Weight gain	0.00	0.00	0.00	0.00	0.00	0.00

In the radiotherapy group, significant articulation defects of multiple phonetic sibilants and mild to moderate speech intelligibility impairment were observed (Table 2). Patients that had adjuvant radiotherapy had absent tongue movements in all directions. Swallowing in liquids was excellent in all patients, but incomplete clearing of the mouth was noted during pureed and solids.

#### Quality-of-life assessment

All six patients completed the European Organization for Research and Treatment of Cancer Quality of Life Questionnaires (EORTC-QLQ) and EORTC-H&N35, which assesses symptoms specific to head and neck cancers.

Patients who did not undergo radiotherapy reported higher overall QOL and better social and sexual functioning (Table 3). They also reported less pain, swallowing problems, speech problems, trouble with social eating and dry mouth (Table 3).

## Discussion

The functional decline observed in these patients caused by radiotherapy is due to its adverse effects on the free flap reconstruction and to the other structures of the oral cavity.

Large reconstructed tongues result in better swallowing function and speech outcomes [5,6]. Flap contraction is a known phenomenon in reconstructive surgery, and during tongue reconstruction it is suggested that an over-correction of 20–30% should be performed to compensate for this volume loss [7]. Radiotherapy following tongue reconstruction results in much greater flap contraction. At one-year post-operatively, magnetic resonance imaging scan analysis estimates the volume loss in irradiated patients is 44.2% (16 cm^3^) compared to 19.8% in the no-radiotherapy group (6.9 cm^3^), which may explain in part the findings of our study [8].

Radiotherapy is also associated with insults to other structures of the oral cavity that explain the poorer results in speech and language in tongue cancer survivors. Salivary gland atrophy and subsequent saliva deficiency causes xerostomia, which was a prevalent complaint in the radiotherapy group in our study, and subsequent difficulties in swallowing [9]. Subcutaneous fibrosis and mucosal oedema also have a direct effect on speech and swallowing and subsequently on the quality of life [10].

Our study highlights the negative effects of post-operative radiotherapy in free flap tongue reconstruction, and plastic surgeons who perform these operations should be aware of these during patient counselling and pre-operative planning. In light of evidence that post-operative radiotherapy reduces flap volume by over 40%, an overcorrection of 1.4 may be appropriate for patients undertaking this adjunct therapy. The authors, however, believe that this overcorrection should be preferred in consideration of the projected prognosis. Patients with projected good prognosis may be the ideal candidates for such an overcorrection, whereas those with poorer prognosis may not be, despite radiotherapy, due to the immediate post-operative adverse effects of a large flap in speech, language and quality of life in the short term (first 12 months after resection). Lastly, the side effects of reduced mobility and subsequent tongue functional impairment may potentially be mitigated by the use of re-innervated flaps, a technique which was not utilised in this study [11]. These flaps provide a sensory and mobility element to the reconstructed neo-tongue and may result in an acceptable quality of life despite the use of radiotherapy.
